# Health behavior associated with liver enzymes among obese Korean adolescents, 2009–2014

**DOI:** 10.1371/journal.pone.0190535

**Published:** 2018-01-17

**Authors:** Eun-young Lee, Hwa Young Choi, Hyunsoon Cho, Bo Hyun Kim, Moran Ki

**Affiliations:** 1 Department of Cancer Control and Population Health, Graduate School of Cancer Science and Policy, National Cancer Center, Goyang, Ilsandong-gu, Goyang, Republic of Korea; 2 Center for Liver Cancer, National Cancer Center, Goyang, Ilsandong-gu, Goyang, Republic of Korea; Chang Gung Memorial Hospital Kaohsiung Branch, TAIWAN

## Abstract

**Aims:**

Obesity is major risk factor for liver health. This study aimed to clarify whether specific health behaviors were associated with liver function in obese adolescents in Korea.

**Methods:**

Based on national school health examination data from 2009 to 2014, 25,142 obese or overweight students were examined for aspartate transaminase and alanine transaminase levels, and health behaviors. Multiple logistic regression was used to calculate the odds ratio for liver enzyme elevation.

**Results:**

Subjects who thought of themselves as “very fat” had a 1.6 times higher odds ratio for liver enzyme elevations than those who thought of themselves as “normal.” Those who consumed fast food 3 to 5 times weekly had 1.3 times higher odds ratio (OR = 1.27, 95% confidence interval = 1.05–1.54) for the elevation of ALT than those who did not consume fast food. Those who took sugar-sweetened beverage 3 to 5 times weekly had 1.2 times higher odds ratio (OR = 1.24, 95% confidence interval = 1.07–1.42) for the elevation of ALT than those who did not take it. Those who played computer game more than 2 hours a day showed 1.1 times higher odds ratio (OR = 1.10, 95% confidence interval = 1.01–1.21) for the elevation of ALT than those who did not.

**Conclusions:**

Specific food item and its frequency of consumption were identified for the positive and negative association with the elevation of liver enzymes. Self-image of body shape, sleeping time and need of help for alcohol or smoking problems also showed substantial association with the elevation.

## Introduction

Obesity is one of the well-known risk factors for liver health, and the prevalence of obesity is steadily increasing in Korean adolescents [1, 2]. This trend is consistent with those in developed countries such as the United States [3, 4]. The prevalence of obesity in Korea was 10.9% among middle and high school students in the 11^th^ Korea Youth Risk Behavior Web-based Survey in 2015[2]. Meanwhile, infection with hepatitis B virus (HBV), which has been a major risk factor for liver health[5], is under control through a national immunization program implemented in 1995 in Korea [6]. Therefore specific study targeting emerging main threat to liver health is needed.

Previous studies regarding the effect of health behavior on liver health have focused on patients with non-alcoholic fatty liver disease (NAFLD). In a 2-year follow-up study by Reinehr et al., a multidisciplinary lifestyle intervention effectively improved NAFLD [7].

Previous studies in Korea in relation to liver health of obese adolescents focused mainly on the association between obesity and metabolic disorders. A strong association between metabolic syndrome and elevated alanine transaminase (ALT) levels in Korean adolescents was found[8, 9]. Regarding the prevalence of elevated liver enzymes, a study by Park et al. established the upper reference limits of aminotransferase levels in a Korean adolescent population [10]. However, the study which sampled general adolescents and focused on the association between specific health behavior and liver function among obese subjects was not easily found.

In the present study, the association between the health behavior of obese adolescents and their liver function was examined through analyzing the results from an annual school health examination, which sampled students across the country. This study aimed to clarify whether specific health behaviors were associated with liver function in obese adolescents.

## Materials and methods

### Sample and data collection

This study was based on the data of the national school health examination, which is conducted every year by Ministry of Education of Korea according to the School Health Act, Article 7. This includes a cross-sectional health examination and a diet and behavior survey. This study used secondary data, of which personal identifiable information was deleted before providing from Ministry of Education, Korea, and ethics approval was not required. Any special access privileges were not needed for data collection and application method for the data is standardized as follows. Firstly, go to the website http://www.open.go.kr and select "Request open" followed by "Apply request" and then log in by joining membership.

Secondly, fill the request format and designate receiving institution as Ministry of education and submit it. Lastly, there will be reply by e-mail.

In this examination, anthropometric data such as height and weight were measured and a survey on health behavior was conducted across all grades, consisting of elementary school (6 years), middle school (3 years), and high school (3 years). The average age for entering elementary school was 7 years. Basic checkups including examination of oral cavities, urine test, and blood pressure was performed only for students in the 1^st^ and 4^th^ year in elementary school (7 and 10 years old) and those in the 1^st^ year in middle and high schools (13 and 16 years old). However, blood tests of fasting glucose, total cholesterol (TC), aspartate transaminase (AST), and ALT were conducted for obese students by Degree of Obesity in 4^th^ year in elementary school, 1^st^ year in middle and high schools only (10, 13 and 16 years old). Degree of Obesity is calculated by the following formula:

DegreeofObesity(%)={(Actualweight−Standardweightbyheight)÷Standardweightbyheight}×100

An HBV infection test was performed for students in the 1^st^ year and in middle school (13 years old) only. This study analyzed health checkup, blood test, and survey data about health behavior for obese students in 4^th^ year in elementary school, 1^st^ year in middle and high schools (10, 13 and 16 years old). (S1 Fig and S2 Fig)

### Study subjects and definition

**Fig 1:**
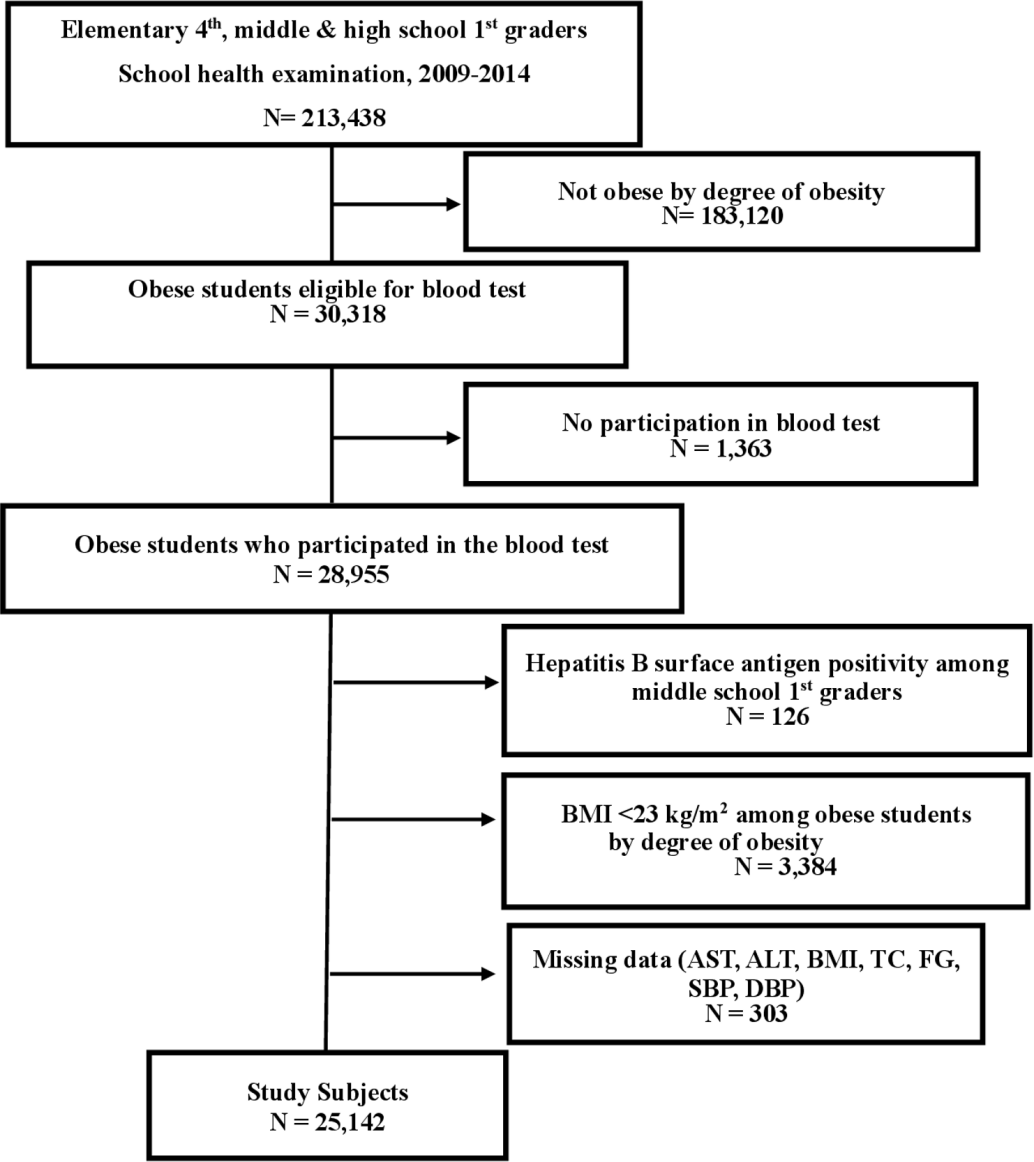
Flow chart of selecting study population.

Among subjects who were obese, those that had positive results for the hepatitis B surface antigen test and were in middle school 1^st^ year were excluded. Those with a body mass index (BMI) <23 kg/m^2^ were also excluded. Subjects with missing AST, ALT, BMI, TC, fasting glucose (FG), systolic blood pressure (SBP), and diastolic blood pressure (DBP) data were also excluded. The number of included subjects was 25,142, consisting of 15,422 boys and 9,720 girls. Please refer to Fig 1 for the inclusion and exclusion criteria. (Fig 1)

Cutoff values for each measurement variable followed the criteria outlined in the school health examination guidelines[1]. For blood pressure, students with ≥130 mmHg (SBP) and ≥80 mmHg (DBP) were categorized as high blood pressure. For TC, values of ≤170 mg/dL, 170 < TC < 200 mg/dL, ≥200 mg/dL were categorized as normal, risky, and hypercholesterolemia, respectively. For FG, <100 mg/dL, 100 < FG < 125 mg/dL, ≥126 mg/dL groups were categorized as normal, blood sugar disorder, and hyperglycemia, respectively. For AST and ALT, students with ≤45 U/L and >45 U/L groups were categorized as normal and suspicious of disease, respectively.

For the metabolic risk profile, four variables consisting of obesity by BMI, hypertension, elevated total cholesterol, and fasting glucose were considered. For obesity, BMI ≥25 was applied. For hypertension, the criteria were SBP ≥130 mmHg and DBP ≥80 mmHg. For elevated total cholesterol, >170 mg/dL was applied. For elevated fasting glucose, ≥was mg/dL was applied.

### Statistical analysis

Differences in categorical variables were evaluated using χ^2^ test. Univariate logistic regression analysis was performed for each variable to check for significant association with the elevation of liver enzymes. A multiple logistic regression was conducted to examine the comprehensive risk for liver enzyme elevation. Statistical analyses were performed with SAS software version 9.3. Statistical significance was defined as *P* < 0.05.

## Results

The study population consisted of 15,422 boys (61%) and 9,720 girls (39%). The majority of the study subjects had a BMI >25 kg/m^2^. Boys had a higher prevalence of liver enzyme elevation than girls. Regarding liver enzymes, there was a higher prevalence of ALT elevation than AST elevation. (Table 1)

**Table 1:** General characteristics of obeseª study subjects.

	Girls (N = 9,720, 39%)		Boys (N = 15,422, 61%)		
Variable	Number	%	Number	%	*p* value ^b^
Age (years) ^c^					< .001
10	1590	16.36	3335	21.62	
13	3473	35.73	6042	39.18	
16	4657	47.91	6045	39.20	
Body mass index (kg/m^2^)					< .001
23≤ <25	2686	27.63	2887	18.72	
≥25	7034	72.37	12535	81.28	
Systolic blood pressure (mmHg)					< .001
<130	9072	93.33	13554	87.89	
≥130	648	6.67	1868	12.11	
Diastolic blood pressure (mmHg)					< .001
<80	8755	90.07	13468	87.33	
≥80	965	9.93	1954	12.67	
Total cholesterol (mg/dL)					
≤170	5269	54.21	8801	57.07	< .001
170–199	3004	30.91	4500	29.18	
≥200	1447	14.89	2121	13.75	
Fasting blood glucose (mg/dL)					
<100	8579	88.26	12635	81.93	< .001
100–125	1065	10.96	2613	16.94	
≥126	76	0.78	174	1.13	
Aspartate transaminase (U/L)					< .001
≤45	9482	97.55	14321	92.86	
>45	238	2.45	1101	7.14	
Alanine transaminase (U/L)					< .001
≤45	9205	94.70	12910	83.73	
>45	515	5.30	2509	16.27	

^a^ all of the study subjects were obese by degree of obesity index.

^b^ Chi-square test was used.

^c^ median age for each school year

BMI, FG, TC, SBP and DBP showed a significant correlation with AST and ALT elevation. (S1 Table) In results of the univariate logistic regression analysis using checkup variables, an increased BMI showed a higher odds ratio for liver enzyme elevation. In the FG, TC, SBP, and DBP groups of higher values showed significantly higher odds ratios for liver enzyme elevation. (S2 Table)

Overall, according to the univariate logistic regression analysis, the boys showed a >3 times higher odds ratio for the elevation of liver enzymes compared to the girls. In relationship with dietary habits, weekly frequency of consuming sugar-sweetened beverages, fast food, and meat showed a positive association with elevation of liver enzymes. Adolescents who ingested sugar-sweetened beverages three to five times per week showed a 1.24 times and 1.44 times higher odds ratio for elevation of AST and ALT, respectively, compared with those who did not participate in this behavior. A positive association was seen between the weekly frequency of fast food consumption and both AST and ALT elevations. Meanwhile, a significant positive association with the consumption of meat was found in ALT elevation only. (Table 2 and S3 Table)

**Table 2:** Relationship between dietary habits and the elevation of liver enzymes^a^.

		Elevation of AST^b^		Elevation of ALT^b^	
Variable	Category	Odds ratio	95% CI	Odds ratio	95% CI
Sex (Referent: Girl)	Boy	3.06	2.66–3.53	3.47	3.15–3.83
Instant noodles/week	1~2 times	1.07	0.93–1.24	1.06	0.96–1.16
(Referent: No)	3~5 times	1.10	0.86–1.40	1.06	0.90–1.26
	Everyday	0.90	0.36–2.22	1.21	0.70–2.10
Beverage/week	1~2 times	1.11	0.94–1.30	1.18	1.06–1.33
(Referent: No)	3~5 times	1.24	1.03–1.50	1.44	1.27–1.64
	Everyday	1.23	0.86–1.75	1.19	0.92–1.54
Fast food/week	1~2 times	1.25	1.11–1.41	1.22	1.12–1.32
(Referent: No)	3~5 times	1.45	1.12–1.88	1.44	1.20–1.72
	Everyday	1.41	0.61–3.24	1.11	0.59–2.08
Meat/week	1~2 times	1.02	0.78–1.32	1.28	1.05–1.56
(Referent: No)	3~5 times	1.10	0.84–1.44	1.43	1.17–1.75
	Everyday	0.96	0.69–1.34	1.34	1.06–1.71
Milk & dairy products/week	1~2 times	1.00	0.77–1.29	0.99	0.83–1.19
(Referent: No)	3~5 times	1.02	0.79–1.32	1.04	0.87–1.24
	Everyday	1.16	0.90–1.49	1.12	0.95–1.34
Fruits/week	1~2 times	0.79	0.62–1.00	1.02	0.85–1.23
(Referent: No)	3~5 times	0.71	0.56–0.90	0.89	0.74–1.06
	Everyday	0.77	0.60–0.99	1.00	0.83–1.20
Vegetables/week	1~2 times	0.68	0.54–0.86	0.91	0.76–1.08
(Referent: No)	3~5 times	0.67	0.53–0.84	0.87	0.73–1.04
	Everyday	0.65	0.51–0.83	0.87	0.75–1.05
Breakfast	Usually take	0.99	0.86–1.14	1.02	0.93–1.13
(Referent: No skip)	Usually skip	1.02	0.84–1.24	1.01	0.88–1.16
	Skip	1.04	0.86–1.26	0.92	0.80–1.05

AST = aspartate transaminase; ALT = alanine transaminase; CI = confidence interval

^a^ univariate logistic regression was used.

^b^ applied criteria was >45U/L.

In the relationship between other health behaviors and the elevation of liver enzymes, students who slept more than 7 hours a day showed higher odds ratio for elevation of AST and ALT compared with those who slept less than 6 hours. Sedentary habits showed a positive association with the elevation of liver enzymes. Subjects who watched TV more than 2 hours a day displayed a 1.53 times higher odds ratio for ALT elevation compared with those who did not. Subjects who played computer games more than 2 hours a day showed a 1.3 times higher odds ratio for ALT elevation compared with those who did not. Watching pornography had a positive association with the elevation of both AST and ALT. In the field of emotional stability and mental stress, the subject’s self-image of his or her body shape showed a positive association. Those who thought of themselves as “very fat” showed a 1.5 times higher odds ratio for the elevation of both AST and ALT compared with those who thought of themselves as “normal”. (Table 3 and S4 Table)

**Table 3:** Relationship between health behaviors in different fields and the elevation of liver enzymes^a^.

			Elevation of AST^b^		Elevation of ALT^b^	
Field	Variable	Category	Odds ratio	95% CI	Odds ratio	95% CI
Physical activity and media use	Average amount of sleep/day (Referent: <6 hours)	6~7 hours	1.15	0.98–1.36	1.09	0.98–1.22
		7~8hours	1.37	1.16–1.61	1.24	1.11–1.39
		>8hours	1.34	1.10–1.63	1.17	1.02–1.34
	≥2 hours/day of computer game (Referent: No)	Yes	1.31	1.16–1.48	1.30	1.20–1.42
	≥3 times a week of exercise^c^ (Referent: No)	Yes	0.84	0.67–1.05	1.07	0.91–1.25
	≥2 hours/day of watching TV^c^ (Referent: No)	Yes	1.76	1.40–2.21	1.53	1.31–1.79
	No. of days a week for exercise^d^(Referent: No)	1~2days	1.18	1.01–1.38	1.19	1.07–1.32
		3~4days	1.29	1.07–1.56	1.26	1.11–1.43
		≥5days	1.19	0.94–1.50	0.98	0.83–1.16
	Watching pornography ^d^ (Referent: No)	Yes	1.24	0.90–1.70	1.42	1.15–1.76
Emotional stability and mental stress	Self-image of body shape (Referent: Normal)	Fat	0.88	0.74–1.04	0.92	0.82–1.04
		Very fat	1.50	1.27–1.78	1.50	1.33–1.68
	Family members' emotional support ^c^ (Referent: No)	Yes	0.97	0.74–1.28	1.02	0.84–1.24
	Feeling hopeless ^b^ (Referent: No)	Yes	1.17	0.71–1.92	1.22	0.86–1.72
	I have a person to discuss worries ^d^ (Referent: No)	Yes	1.09	0.95–1.26	0.90	0.82–0.99
	Worry of problems in family^d^ (Referent: No)	Yes	0.87	0.73–1.03	0.94	0.84–1.05
	Stress for choosing a career path^d^ (Referent: No)	Yes	0.90	0.79–1.03	0.89	0.81–0.98
Personal hygiene and safety consciousness	Washing hands before meal or after coming back home (Referent: No)	Yes	1.05	0.92–1.19	1.03	0.94–1.13
	Brushing teeth ≥twice a day (Referent: No)	Yes	0.72	0.62–0.85	0.75	0.67–0.84
	Wearing safety gear when taking bicycle, skateboard, etc. (Referent: No)	Yes	1.10	0.97–1.26	1.04	0.95–1.13
Substance abuse	A smoking person among people living together ^c^ (Referent: No)	Yes	1.04	0.83–1.30	1.13	0.97–1.32
	A heavy drinker among people living together ^c^ (Referent: No)	Yes	0.84	0.63–1.11	0.96	0.80–1.17
	Need help from expert for alcohol or smoking problems ^d^ (Referent: No)	Yes	1.13	0.64–1.98	1.70	1.22–2.38

AST = aspartate transaminase; ALT = alanine transaminase; CI = confidence interval

^a^ univariate logistic regression was used.

^b^ applied criteria was >45U/L.

^c^ question for elementary school students (7–12 years old)

^d^ question for middle and high school students (13–18 years old)

In the multiple logistic regression analysis results for AST and ALT elevation in all study subjects, more variables were associated with ALT elevation than with AST elevation. Regarding the increase in the frequency of metabolic risk, a rapid increase in the odds ratios for elevation of AST and ALT was found. Subjects who had four metabolic risk profiles showed 7.53 times higher risk for the elevation of ALT compared with those without the risk profiles after adjustment for the effects of other significant variables. An increase in the weekly frequency of fast food intake showed a steady increase in the risk of the elevation of liver enzymes. Those who ate fast food three to five times a week showed a 1.27 times higher risk of the elevation of ALT compared with those who did not, after adjustment for the effects of other significant variables. In regards to self-image of body shape, subjects who thought of themselves as being “very fat” showed a 1.6 times higher risk for the elevation of both AST and ALT compared with those who thought of themselves as “normal,” after adjustment for the effects of other significant variables. (Table 4)

**Table 4:** Risk of the elevation of liver enzymes in all the study subjects using multiple logistic regression model.

Variable (Referent)		AST (>45 U/L)		ALT (>45 U/L)	
	Category	Odds ratio	95% CI	Odds ratio	95% CI
Sex (Girl)	Boy	2.92	2.52–3.38	3.34	3.02–3.70
Age ^a^ (10 years)	13	0.75	0.65–0.87	0.63	0.56–0.70
	16	0.59	0.51–0.69	0.64	0.57–0.71
Frequency of metabolic risk profile ^b^ (0)	1	1.53	1.14–2.04	1.69	1.38–2.06
	2	2.97	2.23–3.95	3.10	2.54–3.79
	3	3.87	2.83–5.31	4.61	3.70–5.76
	4	7.11	4.21–12.01	7.53	5.04–11.24
Fast food/week (No)	1~2 times	1.21	1.07–1.36	1.14	1.05–1.25
	3~5 times	1.36	1.04–1.77	1.27	1.05–1.54
	Everyday	1.28	0.55–2.98	0.92	0.48–1.77
Beverage/week (No)	1~2 times	—		1.09	0.96–1.23
	3~5 times			1.24	1.07–1.42
	Everyday			0.94	0.71–1.23
Fruits/week (No)	1~2 times	—		1.08	0.89–1.30
	3~5 times			0.97	0.80–1.17
	Everyday			1.14	0.93–1.39
Self-image of body shape (Normal)	Fat	0.91	0.77–1.09	0.96	0.85–1.09
	Very fat	1.58	1.33–1.88	1.59	1.41–1.80
≥2 hours/day of computer game (No)	Yes	—		1.10	1.01–1.21

AST = aspartate transaminase; ALT = alanine transaminase; CI = confidence interval

^a^ means median age for each school year

^b^ includes obesity by BMI, hypertension, elevated fasting blood glucose and total cholesterol.

In a separate multiple logistic regression analysis for the elevation of liver enzymes by age and using a separate questionnaire, the gender gap increased in the older age group. Among those who were 13 or 16 years old, boys showed a 4.56 times higher odds ratio for the elevation of ALT than girls, whereas boys who were 10 years old showed a 1.45 times higher odds ratio than girls in the same age group. Among those who were 10 years old, the weekly frequency of eating vegetables and average amount of sleep per day showed a negative association with the elevation of AST and ALT, respectively. On the other hand, among those who were 13 or 16 years old, the number of days a week of exercise showed a negative association with ALT elevation, and need of help from expert for alcohol or smoking showed a positive association with ALT elevation. (Table 5)

**Table 5:** Risk of the elevation of liver enzymes in separate age group ^a^ using multiple logistic regression model.

	Variable (Referent)		AST (>45 U/L)		ALT (>45 U/L)	
Age		Category	Odds ratio	95% CI	Odds ratio	95% CI
10	Sex (Girl)	Boy	1.71	1.29–2.25	1.45	1.21–1.75
	Frequency of metabolic risk profile ^b^ (0)	1	1.65	1.13–2.40	1.67	1.30–2.14
		2	2.61	1.78–3.81	2.49	1.92–3.23
		3	2.77	1.64–4.69	3.42	2.37–4.92
		4	5.03	0.58–43.31	2.07	0.24–17.83
	Vegetables/week (No)	1~2 times	0.63	0.42–0.95	—	
		3~5 times	0.74	0.49–1.12		
		Everyday	0.56	0.36–0.87		
	Meat/week (No)	1~2 times	—		1.49	1.01–2.19
		3~5 times			1.80	1.20–2.70
		Everyday			1.13	0.56–2.28
	Average amount of sleep/day (<6hours)	6~7 hours	—		0.88	0.61–1.27
		7~8 hours			0.72	0.52–0.99
		>8 hours			0.68	0.49–0.95
	Self-image of body shape (Normal)	Fat	1.12	0.78–1.62	0.95	0.75–1.21
		Very fat	1.50	1.04–2.16	1.22	0.95–1.56
	≥2 hours/day of computer game (No)	Yes	—		1.34	1.10–1.63
	≥2 hours/day of watching TV ^c^ (No)	Yes	1.59	1.26–2.01	1.36	1.15–1.61
13 or 16	Sex (Girl)	Boy	3.64	3.01–4.34	4.56	3.96–5.24
	Frequency of metabolic risk profile ^b^ (0)	1	1.54	0.95–2.50	2.45	1.58–3.78
		2	3.28	2.04–5.30	4.95	3.21–7.63
		3	4.31	2.62–7.10	7.35	4.70–11.51
		4	7.34	3.83–14.10	9.23	4.97–17.14
	Beverage/week (No)	1~2 times	—		1.10	0.93–1.29
		3~5 times			1.29	1.08–1.54
		Everyday			1.14	0.82–1.60
	Self-image of body shape (Normal)	Fat	0.84	0.68–1.02	0.98	0.83–1.15
		Very fat	1.58	1.29–1.92	1.75	1.49–2.07
	No. of days a week for exercise ^d^ (No)	1~2days	—		0.96	0.84–1.09
		3~4days			0.94	0.80–1.09
		≥5days			0.69	0.57–0.84
	Need help from expert for alcohol or smoking problems ^d^ (No)	Yes	—		1.45	1.02–2.06

AST = aspartate transaminase; ALT = alanine transaminase; CI = confidence interval

^a^ age group is distinguished by separate questionnaire according to school year.

^b^ includes obesity by BMI, hypertension, elevated fasting blood glucose and total cholesterol.

^c^ question for elementary school students (7–12 years old)

^d^ question for middle and high school students (13–18years old)

## Discussion

This study aimed to clarify whether specific health behaviors were associated with the elevation of liver enzymes among obese adolescents in Korea. First, among all study subjects, self-image of body shape and weekly frequency of ingesting fast food were positively associated with elevated levels of AST and ALT. Weekly frequency of consuming sugar-sweetened beverages and playing more than 2 hours per day of computer games was positively associated with elevated ALT levels.

Second, in a separate analysis by age group according to the questionnaire, differences emerged among significantly associated variables. In the group of 10-year-old subjects, weekly frequency of ingesting vegetables and average amount of sleep showed a negative association, whereas weekly frequency of meat intake and sedentary habits showed positive associations with elevated liver enzymes. On the other hand, in the age group of 13- or 16-year-olds, weekly frequency of consuming sugar-sweetened beverages, and need of help from expert for alcohol or smoking alcohol consumption, and smoking showed a positive association, whereas number of days per week of exercise showed a negative association with elevated liver enzymes.

According to the result of the school health examination conducted in 2014, the average proportion of students with elevated liver enzymes was 12.4% among obese study subjects and the proportion of obesity by BMI percentiles in whole school years consisting of 12 years was 12.9%[1]. Therefore, the estimated prevalence of abnormal liver function in the general adolescent population was around 1.6%. In a cross-sectional study by Park et al. of a sample of the Korean adolescent population during 2007–2009, the prevalence of elevated ALT levels was 6.5%, as a result of different cut-off levels used to define ALT elevation [10]. In the U.S., the prevalence of elevated ALT levels was 7.4% among white adolescents during 1999–2004[11].

For dietary habits, a significant association with consumption of instant noodles and liver enzyme elevation was not found in this study. In a study done in Japan targeting male workers, however, habitual ingestion of instant noodles was associated with elevated ALT and gamma-glutamyl transferase (GGT) [12]. In a study by Ma et al., sugar-sweetened beverage consumption was positively associated with ALT levels [13]. Regarding NAFLD as an advanced consequence of lowered liver function, a prospective cohort study of dietary habits by Oddy et al. indicated that a Western dietary pattern at 14 years of age in a general population sample was associated with an increased risk of NAFLD at 17 years of age, particularly in obese adolescents [14]. For more detailed data concerning nutriments, however, a case control study by Gibson et al. compared pediatric NAFLD patients and obese children without liver disease with respect to their dietary habits. No differences in macro- or micronutrient intake were observed between children with NAFLD and obese controls [15].

In the present study, a negative association was evident between brushing teeth more than twice a day and elevation of ALT. This finding is in line with those of previous studies dealing with oral health and liver enzymes. A cross-sectional study by Wiener et al. indicated the association of ALT with periodontitis [16]. In another study by Ahmad et al., the association between periodontal condition and the combination of elevated ALT and MetS was confirmed in males [17].

A study by Claris Martins, et al. reported that sedentary behavior was positively associated with ALT, while moderate-to-vigorous physical activity was negatively associated with ALT levels [18]. In the present study, among subjects with sedentary habits, a positive association with elevated liver enzymes was evident. On the other hand, a case control study by Gibson et al., which compared the physical activity of pediatric NAFLD patients and obese children without liver disease, indicated that children with NAFLD were more physically active than their obese counterparts. The authors interpreted this to mean that the greater awareness of the importance of physical activity may be a consequence of the NAFLD diagnosis [15].

According to a review of Vere et al., stress has been identified in recent years as an important factor in the progression and outcome of several important liver pathologies [19]. In the present study, for subjects 13 or 16 years of age who had persons with whom to discuss stressors, a lower odds ratio was noted for ALT elevation. For those subjects who thought that they were “very fat”, a higher odds ratio for AST and ALT elevation were evident in all study subjects.

The strength of this study is the representativeness of the sample of general adolescents in Korea. Adolescents were sampled from across the country, and they were beneficiaries of the national HBV immunization program. Limitations of this study include that both anthropometric values and questionnaire data were recorded only once. Therefore, the long-term effect of these behaviors on liver health could not be observed. Further study including long-term follow-up is needed.

In conclusion, specific health behaviors associated with liver function among obese adolescents differed by age and liver enzyme. In general, frequent ingestion of fast food and sugar-sweetened beverages and fruits, and playing computer games more than 2 hours a day increased the risk of elevated liver enzymes. And self-image of body shape showed substantial and constant association with the elevation. The strength of risk for elevated liver enzymes through health behaviors was relatively weak compared with that by the metabolic risks that are accompanied by obesity. Therefore, preventing obesity is an effective way of maintaining liver health in adolescents. Various strategies including family-based behavioral therapy, considered to be a gold standard for childhood weight loss, may be considered for current and potentially obese students. Findings of this study can act as a basis for obese adolescents to choose better health behaviors in order to protect their liver health.

## Supporting information

S1 Fig Outline of school health examination. (DOCX)

S2 Fig Details of health check up. (DOCX)

S1 Table Correlation between health checkup variables and liver enzymes. (DOCX)

S2 Table Relationship between health checkup variables and the elevation of liver enzymes. (DOCX)

S3 Table Proportions of variables between dietary habits and the elevation of liver enzymes. (DOCX)

S4 Table Proportions of variables between health behaviors in different fields and the elevation of liver enzymes. (DOCX)

## Abbreviations

AST: aspartate transaminase
ALT: alanine transaminase
BMI: body mass index
OR: odds ratio
CI: confidence interval
HBV: hepatitis B virus
NAFLD: non-alcoholic fatty liver disease
SBP: systolic blood pressure
DBP: diastolic blood pressure
GGT: gamma-glutamyl transferase
