# Supplementary material for: Health behavior associated with liver enzymes among obese Korean adolescents, 2009–2014
Source: PLoS One. 2018 Jan 17;13(1):e0190535. doi: 10.1371/journal.pone.0190535 (PMC5771561; doi:10.1371/journal.pone.0190535)
Supplement: S1 Fig — (DOCX) [file pone.0190535.s001.docx]

| Category | Checklist | Applicable school year ^a^ |
| --- | --- | --- |
| Physical development | Height, weight | Elementary school 1^st^ through High school 3^rd^ grade |
| Health survey | Health behavior, life style by questionnaire | Elementary school 1^st^ through High school 3^rd^ grade |
| Health checkup | Musculoskeletal system, eye, ear, nose, neck, skin, oral cavity, pathology laboratory test, etc. | - Elementary school 1^st^ and 4^th^ grade  - Middle school 1^st^ grade  - High school 1^st^ grade |
| Physical fitness | Physical capacity test | Elementary school 5^th^ through high school 3^rd^ grade |

**S1 Fig. Outline of school health examination**

a Applicable school year bases on the school system consisting of elementary school(6 years), middle school(3 years), and high school(3 years).
