# Supplementary material for: Health behavior associated with liver enzymes among obese Korean adolescents, 2009–2014
Source: PLoS One. 2018 Jan 17;13(1):e0190535. doi: 10.1371/journal.pone.0190535 (PMC5771561; doi:10.1371/journal.pone.0190535)
Supplement: S2 Fig — (DOCX) [file pone.0190535.s002.docx]

**S2 Fig. Details of health check up**

| Category | Applicable school year ^a^ (grade) | | | |
| --- | --- | --- | --- | --- |
|  | Elementary school 1st | Elementary school 4th | Middle school 1st | High school 1st |
| Basis | Musculoskeletal system, eye, ear, nose, neck, skin, oral cavity, urine test,  blood pressure | | | |
| Pathology laboratory test | Blood type | Color sense test | -Color sense test  -Hepatitis B virus infection test  -Tuberculosis test | -Tuberculosis test  -Anemia test(Girls) |
|  |  | Obese ^b^ students only  (Blood glucose, total cholesterol, AST, ALT) | | |
| Health survey | -Health behaviors including dietary habits, life style, sleep, physical activity, etc.  -Need of health related consultation | | | |

a Applicable school year bases on the school system consisting of elementary school (6 years), middle school (3 years), and high school (3 years).

b obese by degree of obesity index
