# Supplementary material for: Health behavior associated with liver enzymes among obese Korean adolescents, 2009–2014
Source: PLoS One. 2018 Jan 17;13(1):e0190535. doi: 10.1371/journal.pone.0190535 (PMC5771561; doi:10.1371/journal.pone.0190535)
Supplement: S1 Table — (DOCX) [file pone.0190535.s003.docx]

| **S1 Table. Correlation between health checkup variables and liver enzymes** | | | | | | |
| --- | --- | --- | --- | --- | --- | --- |
|  | AST ^a^ | | | ALT ^a^ | | |
|  | N | coefficient | *p* value | N | coefficient | *p* value |
| Body mass index | 25141 | 0.10 | <.0001 | 25122 | 0.19 | <.0001 |
| Fasting glucose | 25141 | 0.03 | <.0001 | 25122 | 0.06 | <.0001 |
| Total cholesterol | 25141 | 0.11 | <.0001 | 25122 | 0.15 | <.0001 |
| Systolic blood pressure | 25141 | 0.02 | 0.0003 | 25122 | 0.07 | <.0001 |
| Diastolic blood pressure | 25141 | 0.02 | 0.0004 | 25122 | 0.06 | <.0001 |
| N=number; AST=aspartate transaminase; ALT= alanine transaminase | | | | |  |  |
| ^a^ log_10_[AST], log_10_[ALT] was used. | | | | |  |  |
