# Supplementary material for: Health behavior associated with liver enzymes among obese Korean adolescents, 2009–2014
Source: PLoS One. 2018 Jan 17;13(1):e0190535. doi: 10.1371/journal.pone.0190535 (PMC5771561; doi:10.1371/journal.pone.0190535)
Supplement: S2 Table — (DOCX) [file pone.0190535.s004.docx]

|  |  |  |  |  |  |
| --- | --- | --- | --- | --- | --- |
| **S2 Table. Relationship between health checkup variables and the elevation of liver enzymes** ^a^ | | | | | |
|  |  | Elevation of AST ^b^ | | Elevation of ALT ^b^ | |
| Variable (unit, referent) | Category | Odds ratio | 95% CI | Odds ratio | 95% CI |
| Age ^c^(years,10) | 13 | 0.81 | 0.71-0.94 | 0.71 | 0.64-0.78 |
|  | 16 | 0.63 | 0.54-0.72 | 0.71 | 0.64-0.78 |
| Body mass index (kg/㎡, 23.0– 24.9) | ≥25.0 | 1.76 | 1.50-2.05 | 2.02 | 1.81-2.26 |
| Fasting glucose (mg/dL, <100) | 100–125 | 1.29 | 1.12-1.49 | 1.34 | 1.21-1.48 |
|  | ≥126 | 2.97 | 2.06-4.28 | 2.97 | 2.24-3.93 |
| Total cholesterol (mg/dL, ≤170) | 171–199 | 1.67 | 1.47-1.90 | 1.58 | 1.45-1.73 |
|  | ≥200 | 3.27 | 2.85-3.75 | 2.61 | 2.37-2.89 |
| Systolic blood pressure (mmHg,<130) | ≥130 | 1.68 | 1.44-1.96 | 1.92 | 1.73-2.14 |
| Diastolic blood pressure (mmHg,<80) | ≥80 | 1.55 | 1.33-1.80 | 1.66 | 1.50-1.84 |
| AST=aspartate transaminase; ALT=alanine transaminase; CI=confidence interval | | | | |  |
| ^a^ univariate logistic regression was used. | |  |  |  |  |
| ^b^ applied criteria was >45U/L. |  |  |  |  |  |
| ^c^ median age for each school year |  |  |  |  |  |
