# Supplementary material for: Health behavior associated with liver enzymes among obese Korean adolescents, 2009–2014
Source: PLoS One. 2018 Jan 17;13(1):e0190535. doi: 10.1371/journal.pone.0190535 (PMC5771561; doi:10.1371/journal.pone.0190535)
Supplement: S3 Table — (DOCX) [file pone.0190535.s005.docx]

| **S3 Table. Proportions of variables between dietary habits and the elevation of liver enzymes** | | | | | |
| --- | --- | --- | --- | --- | --- |
|  |  | Elevation of AST^a^ | | Elevation of ALT^a^ | |
| Variable | Category | Number | Percentage^b^ | Number | Percentage^b^ |
| Instant noodles/week | No | 245 | 18.69 | 562 | 18.97 |
|  | 1–2 times | 969 | 73.91 | 2182 | 73.64 |
|  | 3–5 times | 92 | 7.02 | 204 | 6.88 |
|  | Everyday | 5 | 0.38 | 15 | 0.51 |
| Beverage/week | No | 193 | 14.68 | 412 | 13.86 |
|  | 1–2 times | 787 | 59.85 | 1771 | 59.59 |
|  | 3–5 times | 297 | 22.59 | 711 | 23.92 |
|  | Everyday | 38 | 2.89 | 78 | 2.62 |
| Fast food/week | No | 440 | 33.49 | 1019 | 34.31 |
|  | 1–2 times | 796 | 60.58 | 1779 | 59.90 |
|  | 3–5 times | 72 | 5.48 | 161 | 5.42 |
|  | Everyday | 6 | 0.46 | 11 | 0.37 |
| Meat/week | No | 63 | 4.79 | 115 | 3.87 |
|  | 1–2 times | 719 | 54.68 | 1613 | 54.29 |
|  | 3–5 times | 450 | 34.22 | 1038 | 34.94 |
|  | Everyday | 83 | 6.31 | 205 | 6.90 |
| Milk & dairy products/week | No | 73 | 5.55 | 167 | 5.62 |
|  | 1–2 times | 328 | 24.94 | 750 | 25.24 |
|  | 3–5 times | 374 | 28.44 | 868 | 29.21 |
|  | Everyday | 540 | 41.06 | 1187 | 39.94 |
| Fruits/week | No | 84 | 6.38 | 152 | 5.11 |
|  | 1–2 times | 486 | 36.93 | 1123 | 37.79 |
|  | 3–5 times | 444 | 33.74 | 1000 | 33.65 |
|  | Everyday | 302 | 22.95 | 697 | 23.45 |
| Vegetables/week | No | 91 | 6.91 | 159 | 5.35 |
|  | 1–2 times | 451 | 34.27 | 1041 | 35.02 |
|  | 3–5 times | 446 | 33.89 | 1010 | 33.97 |
|  | Everyday | 328 | 24.92 | 763 | 25.66 |
| Breakfast | No skip | 798 | 60.68 | 1812 | 61.01 |
|  | Usually take | 264 | 20.08 | 618 | 20.81 |
|  | Usually skip | 123 | 9.35 | 276 | 9.29 |
|  | Skip | 130 | 9.89 | 264 | 8.89 |
| AST=aspartate transaminase; ALT=alanine transaminase; CI=confidence interval | | | | |  |
| ^a^ applied criteria was >45U/L. | |  |  |  |  |
| ^b^ means percentage among those with elevated liver enzymes. | | | |  |  |
