# Supplementary material for: Health behavior associated with liver enzymes among obese Korean adolescents, 2009–2014
Source: PLoS One. 2018 Jan 17;13(1):e0190535. doi: 10.1371/journal.pone.0190535 (PMC5771561; doi:10.1371/journal.pone.0190535)
Supplement: S4 Table — (DOCX) [file pone.0190535.s006.docx]

**S4 Table. Proportions of variables between health behaviors in different fields and the elevation of liver enzymes ^a^**

|  |  |  | Elevation of AST^b^ | | Elevation of ALT^b^ | |
| --- | --- | --- | --- | --- | --- | --- |
| Field | Variable | Category | Number | Percentage^e^ | Number | Percentage^e^ |
| Physical activity and media use | Average amount of sleep/day | <6 hours | 218 | 16.60 | 531 | 17.89 |
|  |  | 6–7 hours | 454 | 34.58 | 1047 | 35.28 |
|  |  | 7–8hours | 444 | 33.82 | 972 | 32.75 |
|  |  | >8hours | 197 | 15.00 | 418 | 14.08 |
|  | ≥2 hours/day of computer game | No | 907 | 69.08 | 2063 | 69.63 |
|  |  | Yes | 406 | 30.92 | 900 | 30.37 |
|  | ≥3 times a week of exercise^c^ | No | 168 | 52.34 | 338 | 46.88 |
|  |  | Yes | 153 | 47.66 | 383 | 53.12 |
|  | ≥2 hours/day of watching TV^c^ | No | 133 | 40.67 | 329 | 44.70 |
|  |  | Yes | 194 | 59.33 | 407 | 55.30 |
|  | No. of days a week for exercise^d^ | No | 280 | 28.57 | 652 | 29.32 |
|  |  | 1–2days | 407 | 41.53 | 944 | 42.45 |
|  |  | 3–4days | 191 | 19.49 | 429 | 19.29 |
|  |  | ≥5days | 102 | 10.41 | 199 | 8.95 |
|  | Watching pornography ^d^ | No | 941 | 95.73 | 2120 | 95.28 |
|  |  | Yes | 42 | 4.27 | 105 | 4.72 |
| Emotional stability and mental stress | Self-image of body shape | Normal | 184 | 13.98 | 412 | 13.87 |
|  |  | Fat | 521 | 39.59 | 1234 | 41.53 |
|  |  | Very fat | 611 | 46.43 | 1325 | 44.60 |
|  | Family members' emotional support ^c^ | No | 72 | 21.75 | 156 | 21.05 |
|  |  | Yes | 259 | 78.25 | 585 | 78.95 |
|  | Feeling hopeless ^b^ | No | 313 | 94.56 | 699 | 94.46 |
|  |  | Yes | 18 | 5.44 | 41 | 5.54 |
|  | I have a person to discuss worries ^d^ | No | 266 | 27.06 | 682 | 30.62 |
|  |  | Yes | 717 | 72.94 | 1545 | 69.38 |
|  | Worry of problems in family^d^ | No | 815 | 82.91 | 1819 | 81.72 |
|  |  | Yes | 168 | 17.09 | 407 | 18.28 |
|  | Stress for choosing a career path^d^ | No | 636 | 65.03 | 1444 | 65.1 |
|  |  | Yes | 342 | 34.97 | 774 | 34.9 |
| Personal hygiene and safety consciousness | Washing hands before meal or after coming back home | No | 308 | 23.53 | 705 | 23.85 |
|  |  | Yes | 1001 | 76.47 | 2251 | 76.15 |
|  | Brushing teeth ≥twice a day | No | 194 | 14.78 | 416 | 14.01 |
|  |  | Yes | 1119 | 85.22 | 2548 | 85.96 |
|  | Wearing safety gear when taking bicycle, skateboard, etc. | No | 990 | 75.46 | 2268 | 76.6 |
|  |  | Yes | 322 | 24.54 | 693 | 23.4 |
| Substance abuse | A smoking person among people living together ^c^ | No | 163 | 49.24 | 353 | 47.64 |
|  |  | Yes | 168 | 50.76 | 388 | 52.36 |
|  | A heavy drinker among people living together ^c^ | No | 268 | 80.97 | 583 | 78.78 |
|  |  | Yes | 63 | 19.03 | 157 | 21.22 |
|  | Need help from expert for alcohol or smoking problems ^d^ | No | 728 | 98.25 | 1709 | 97.55 |
|  |  | Yes | 13 | 1.75 | 43 | 2.45 |
| AST= aspartate transaminase; ALT=alanine transaminase; CI= confidence interval | | | | |  |  |
| ^a^ univariate logistic regression was used. | |  |  |  |  |  |
| ^b^ applied criteria was >45U/L. | |  |  |  |  |  |
| ^c^ question for elementary school students (7-12 years old) | | |  |  |  |  |
| ^d^ question for middle and high school students (13-18 years old) | | | |  |  |  |
| e means percentage among those with elevated liver enzymes. | | | |  |  |  |
